# Advanced intrahepatic cholangiocarcinoma successfully treated with combined immunotherapy: focusing on the tumor immune microenvironment

**DOI:** 10.1007/s12328-025-02113-2

**Published:** 2025-03-28

**Authors:** Mayu Omoto, Katsutoshi Sugimoto, Yutaka Kurebayashi, Tatsuya Kakegawa, Hiroshi Takahashi, Takuya Wada, Hirohito Takeuchi, Toshitaka Nagao, Yuichi Nagakawa, Takao Itoi

**Affiliations:** 1 Department of Gastroenterology and Hepatology, Tokyo Medical University, 6-7-1 Nishishinjuku, Shinjuku-ku, Tokyo, 1600023 Japan; 2 Department of Pathology, Keio University School of Medicine, Tokyo, Japan; 3 Department of Anatomic Pathology, Tokyo Medical University, Tokyo, Japan; 4 Department of Gastrointestinal and Pediatric Surgery, Tokyo Medical University, Tokyo, Japan

**Keywords:** Conversion surgery, Immune checkpoint inhibitor, Tumor-infiltrating lymphocytes

## Abstract

A 61-year-old female patient with advanced intrahepatic cholangiocarcinoma diagnosed based on imaging and tumor biopsy findings was treated with combination therapy comprising gemcitabine, cisplatin, and durvalumab. After eight cycles of therapy comprising gemcitabine, cisplatin, and durvalumab and two subsequent cycles of maintenance immunotherapy, significant tumor shrinkage enabled conversion surgery with R0 resection. The tumor immune microenvironment has a critical role in predicting the efficacy of combined immunotherapy in some types of cancer; however, its role in advanced intrahepatic cholangiocarcinoma remains largely unclear. In the current case, the tumor exhibited increased infiltration of CD8 T cells before treatment, and significant increase in CD8 T-cell infiltration, decrease in Treg/CD8 ratio, and development of tertiary lymphoid structures were observed after treatment. Pretreatment tumor immune microenvironment analyses may predict treatment outcomes and optimize strategies for advanced intrahepatic cholangiocarcinoma. Therapy comprising gemcitabine, cisplatin, and durvalumab and immune-based approaches may enhance personalized medicine for patients with advanced intrahepatic cholangiocarcinoma.

## Introduction

Intrahepatic cholangiocarcinoma (ICC) is a malignant liver tumor that originates from secondary bile ducts and their branches in the liver. Furthermore, ICC is the second most common malignant liver tumor and accounts for 10–15% of all primary liver cancers [1]. Surgical resection is the preferred treatment for ICC; however, because of its subtle onset, most patients have lymph node or distant metastases at the time of diagnosis and cannot undergo surgical resection [2].

Recently, TOPAZ-1, an international phase III trial, was conducted to evaluate the efficacy and safety of gemcitabine plus cisplatin (GC) combined with durvalumab (GCD), an anti-PD-L1 human monoclonal antibody immune checkpoint inhibitor (ICI), as therapy for patients with untreated advanced biliary tract cancer. Among the 685 participants, 383 (55.9%) had ICC [3]. The GCD therapy group experienced significantly improved overall survival (the primary endpoint) compared to that of the GC group (median survival time, 12.8 months vs. 11.5 months; hazard ratio, 0.80; 95% confidence interval, 0.66–0.97).

Based on these findings, GCD therapy is recommended as first-line chemotherapy for ICC according to the European Association for the Study of the Liver–International Liver Cancer Association guidelines, which are widely referenced in Western countries. Similarly, in Japan, durvalumab was approved for clinical use for the treatment of unresectable biliary tract cancer in December 2022. However, the objective response rate, including the complete response rate (2.1%) and partial response rate (24.8%), remains suboptimal at 26.7%.

We present a case of advanced-stage ICC with remarkable tumor shrinkage attributable to GCD therapy. Ultimately, conversion surgery was performed and resulted in a tumor-free state. An understanding of the tumor immune microenvironment (TIME) is crucial in the era of immunotherapy; therefore, we specifically focused on the TIME of the present case.

## Case report

In August 2023, a 61-year-old female patient presented to her general physician with right-side abdominal pain that had persisted for more than 2 weeks. Plain computed tomography (CT) revealed a liver mass; therefore, the patient was referred to our hospital. The patient did not have a relevant medical history. Laboratory findings at the initial presentation indicated slight liver dysfunction (aspartate aminotransferase, 34 IU/L; alanine aminotransferase, 33 IU/L; gamma-glutamyl transferase, 46 mg/dL) and increased tumor marker levels (carcinoembryonic antigen, 5.7 mg/dL; carbohydrate antigen 19–9, 55.9 mg/dL).

**Fig. 1:**
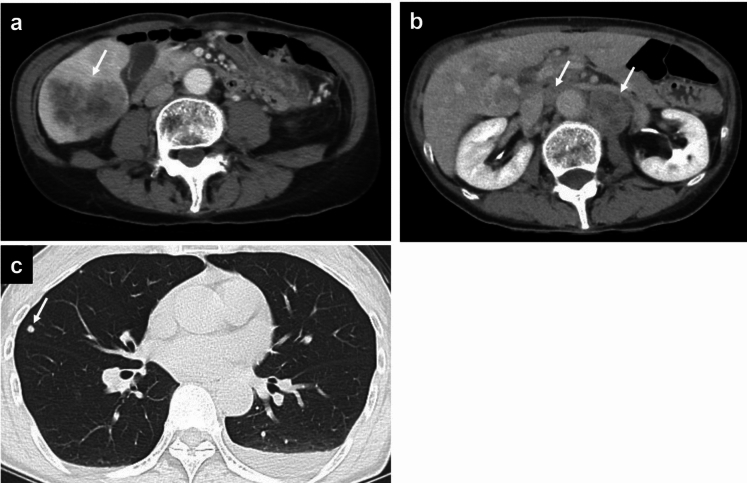
Abdominal and chest contrast-enhanced computed tomography (CT). **a** A 6-cm-diameter liver tumor in segment 6 is observed in the portal venous phase with contrast-enhanced CT (arrow). **b** A swollen para-aorta lymph node is observed in the equilibrium phase with contrast-enhanced CT (arrows). **c** Lung metastases are observed with chest CT (arrow)

**Fig. 2:**
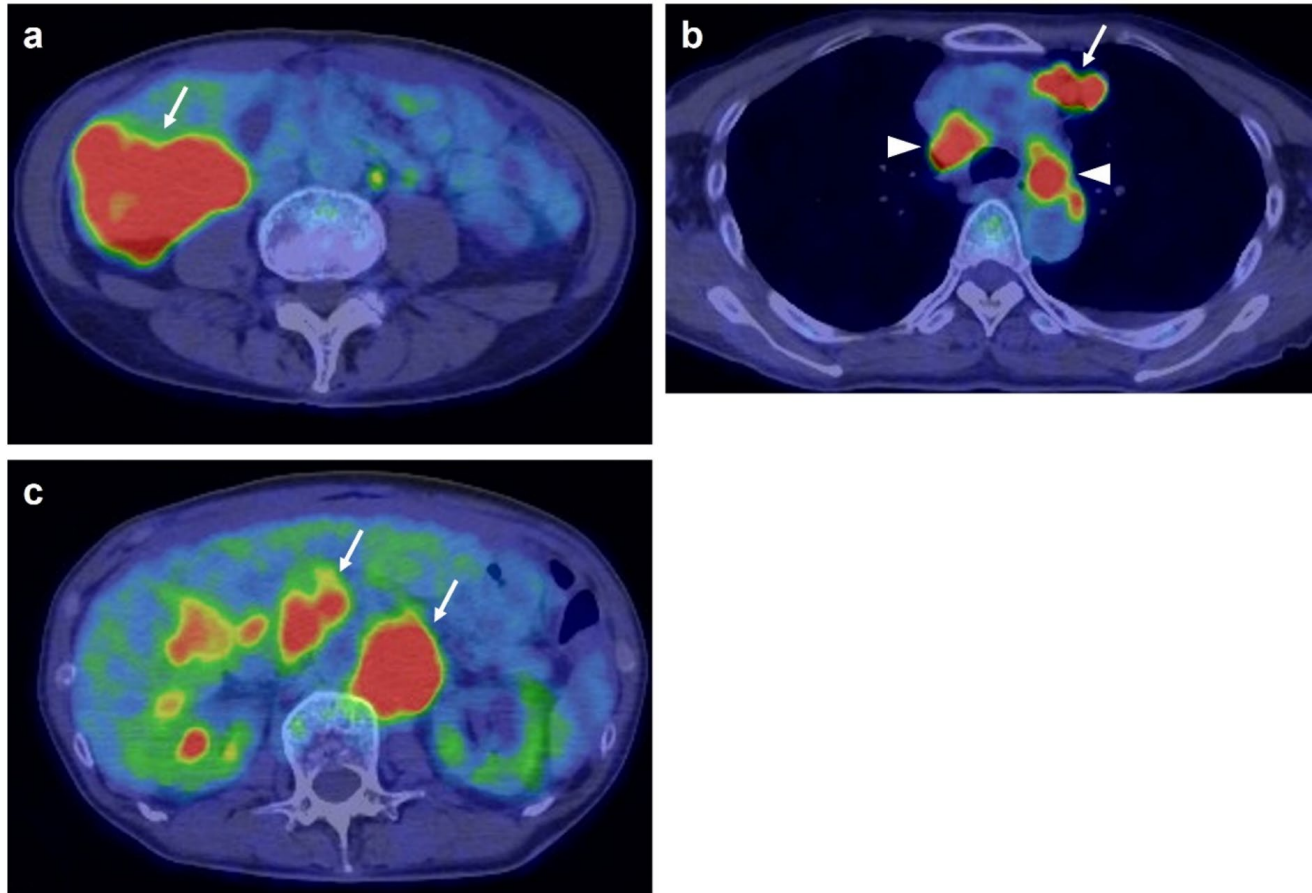
Whole-body positron emission tomography with 2-deoxy-2-[fluorine-18] fluoro-D-glucose combined with computed tomography (18F-FDG PET/CT). Hypermetabolic lesions in the liver (a: arrow), lungs (b: arrow), and lymph nodes (b: arrowheads; c: arrows) are observed

**Fig. 3:**
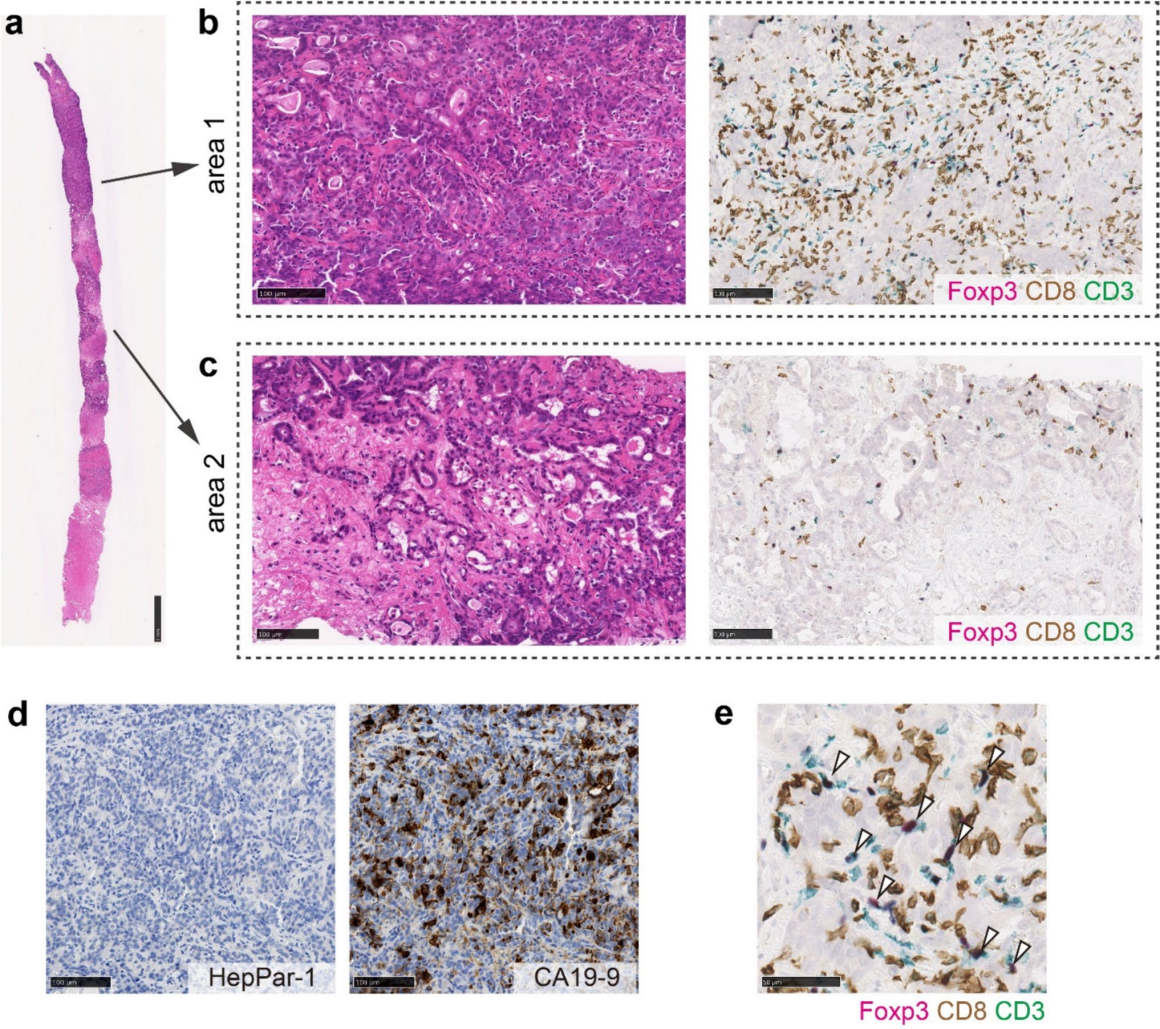
Histopathology and tumor immune microenvironment of the tumor specimen before treatment. **a** Loupe image of the biopsy specimen. Scale bar: 1 mm. The specimen contains an area of (**b**) moderately to poorly differentiated adenocarcinoma proliferating in a cribriform pattern and solid nests with higher increased T-cell infiltration (area 1, total T cell: 929 cells/mm^2^, CD8 T cell: 687 cells/mm^2^, Treg/CD8 ratio: 0.27) and an area of (**c**) moderately differentiated tubular adenocarcinoma associated with fibrotic stroma and lower lymphocytic infiltration (area 2, total T cell: 372 cells/mm^2^, CD8 T cell: 230 cells/mm^2^, Treg/CD8 ratio: 0.35). Scale bar: 100 μm. Arrow heads indicate Foxp3^+^CD3^+^CD8^−^ Treg cells. **d** Inset of (**b**) is shown. Infiltrating T cells are mainly comprised CD8 T cells. Scale bar: 50 μm. **e** Immunohistochemistry for Hep-par1 and CA19-9. Scale bar: 100 μm

Abdominal contrast-enhanced CT (CECT) revealed multiple liver lesions. The largest lesion, which was located in liver segment 6, had a 60-mm diameter and exhibited ring enhancement in the arterial phase and delayed contrast enhancement in the equilibrium phase. In addition, multiple enlarged abdominal lymph nodes were observed, suggesting lymph node metastases. Chest CT revealed multiple lung lesions consistent with metastatic disease (Fig. 1a–c). Positron emission tomography (PET) with 2-deoxy-2-[fluorine-18] fluoro-D-glucose combined with CT (18F-FDG PET/CT) revealed hypermetabolic lesions in the liver, lungs, and lymph nodes (Fig. 2a–c). To establish a definitive diagnosis, a percutaneous ultrasound-guided biopsy of the liver tumor was performed. Pathological examination (Fig. 3 a–d) confirmed the diagnosis of moderately to poorly differentiated ICC. The tumor was positive for CA19-9 and negative for Hep-par1 (Fig. 3d).

Genomic profiling using next-generation sequencing identified mutations in *TP53* and *DNMT*, encoding DNA methyltransferase, as actionable genetic abnormalities. However, no druggable genetic abnormalities, including microsatellite instability-high (MSI-H), were detected. The tumor mutation burden was calculated as 1.26 mutations per megabase, indicating a relatively low tumor mutational burden.

*TIME status in pre-treatment tumor biopsy*: The immune microenvironment of tumor was examined by multiplex immunohistochemistry as we had previously reported with hepatocellular carcinoma (HCC) samples [4]. Since the T-cell infiltration data are not currently available for advanced ICC, we provisionally compared the results with data from advanced HCC [4]. The tumor contained areas of moderately to poorly differentiated adenocarcinoma proliferating in a cribriform pattern and solid nests with higher lymphocytic infiltration (area 1, Fig. 3b), moderately differentiated tubular adenocarcinoma with fibrotic stroma and lower lymphocytic infiltration (area 2, Fig. 3c), and necrosis. The infiltrating T cells were mainly CD8 T cells (total T cell: 929 cells/mm^2^, CD8 T cell: 687 cells/mm^2^ in area 1, and total T cell: 372 cells/mm^2^, CD8 T cell: 230 cells/mm^2^ in area 2), which were much higher the median of those observed in advanced HCC cases (median of total T cell: 118 cells/mm^2^, median of CD8 T cell: 70 cells/mm^2^) (4). The frequency of regulatory T cells (Treg) evaluated as Treg/CD8 ratio (0.27 in area 1 and 0.32 in area 2) was around the median of Treg/CD8 ratio (0.30) that we have observed in a cohort of advanced HCC (4) (Fig. 3e).

**Fig. 4:**
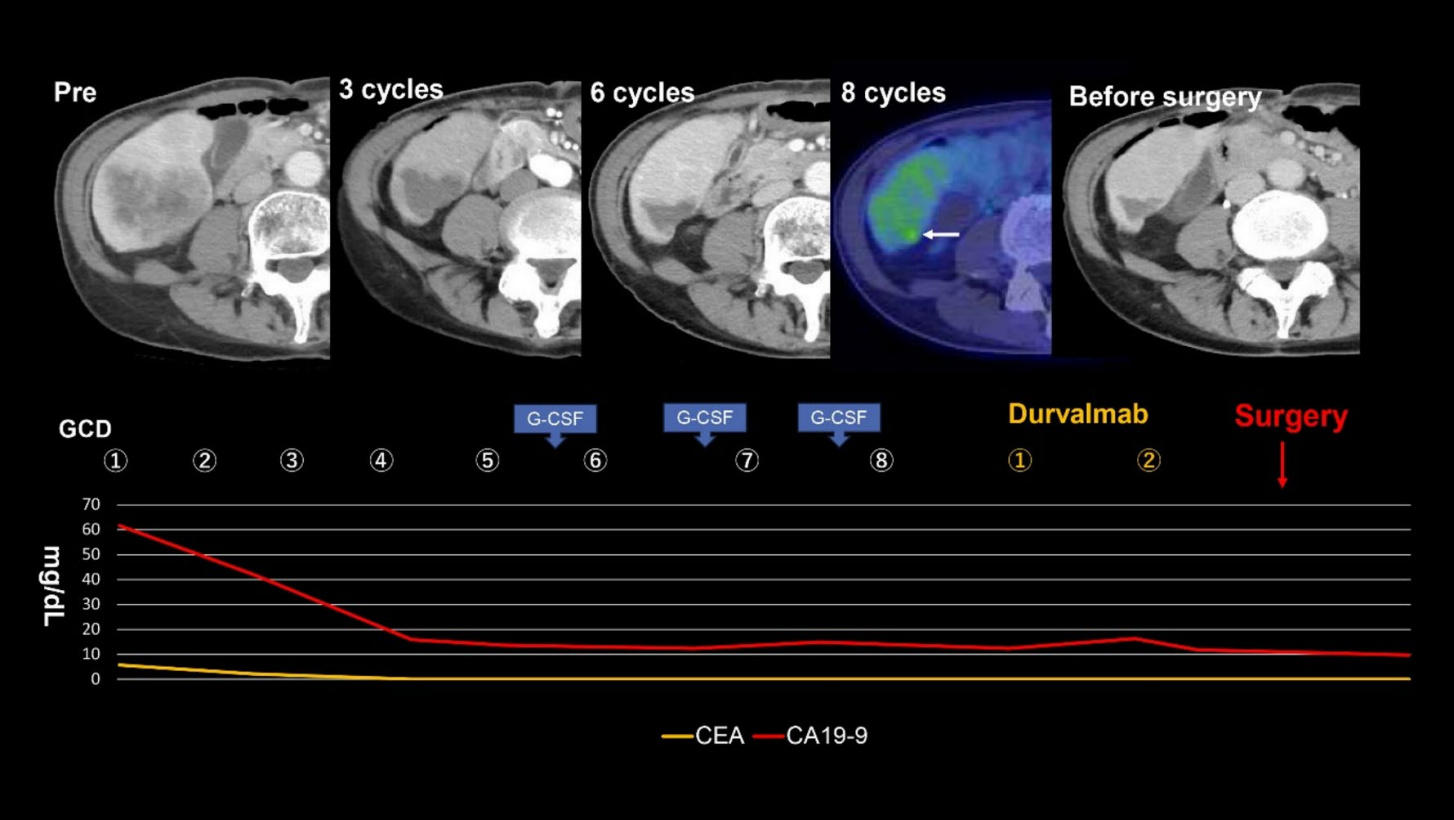
Clinical course including combined immunotherapy, surgery, and radiological examination schedules and tumor marker findings. CA19-9, carbohydrate antigen 19–9; CEA, carcinoembryonic antigen; GCD, gemcitabine, cisplatin, and durvalumab; G-GSF, granulocyte colony-stimulating factor

Based on the findings, GCD therapy (gemcitabine, 1000 mg/m^2^, intravenous [IV] infusion, days 1 and 8; cisplatin, 25 mg/m^2^, IV infusion, days 1 and 8; and durvalumab, 1500 mg/body, IV infusion, day 1) was initiated (Fig. 4). After three cycles of GCD therapy, CECT revealed a marked reduction in the tumor size and loss of contrast enhancement, and lung and lymph node metastases completely disappeared. After six cycles, CECT demonstrated further tumor shrinkage, and lung and lymph node metastases still completely disappeared. After eight cycles, 18F-FDG PET/CT revealed slight residual metabolic activity in the liver tumor, and lung and lymph node metastases did not exhibit FDG uptake.

**Fig. 5:**
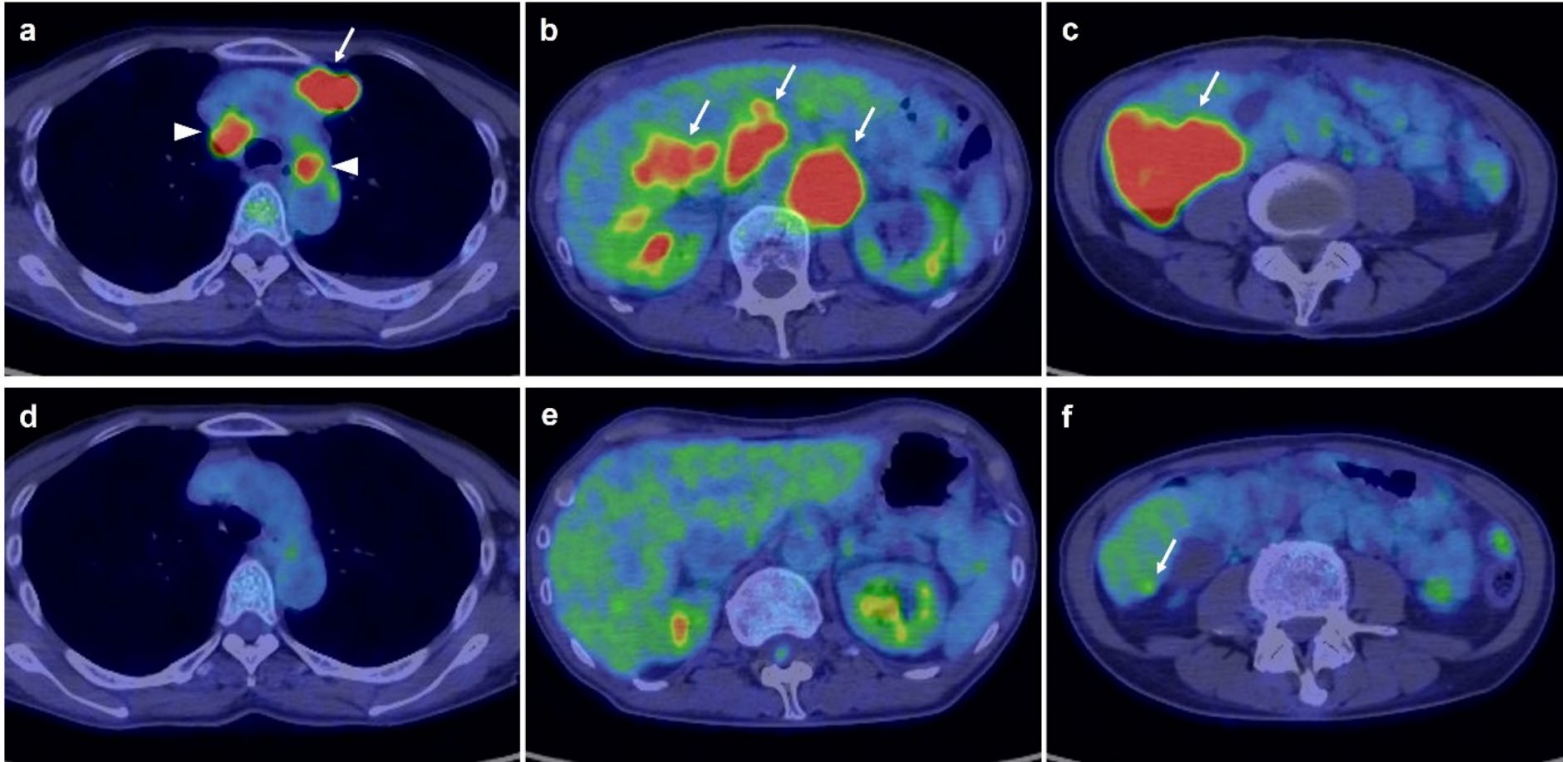
Temporal changes of PET–CT images in lesions before and after gemcitabine plus cisplatin combined with durvalumab (GCD) therapy. (**a**–**c**) pretreatment PET–CT images, (**d**–**f**) posttreatment (after 8 cycles) PET–CT images. **a** Hypermetabolic lesions in lung (arrow) and mediastinum lymph nodes (arrow heads) are identified. **b** Hypermetabolic lesions in abdominal lymph nodes (arrows) are identified. **c** Hypermetabolic lesion in liver (SUV-max value: 12.73) is identified. **d** No hypermetabolic lesions in lung and mediastinum lymph nodes are identified. **e** No hypermetabolic lesions in abdominal lymph nodes are identified. **f** Slight hypermetabolic lesion in liver (SUV-max value: 3.33) is identified (arrow)

After completing eight cycles of GCD therapy, maintenance therapy with durvalumab monotherapy was initiated according to the manufacturer’s recommendations. After two cycles of maintenance therapy, the tumor marker levels returned to normal limits, and a partial treatment response was observed. Although grade 3 neutropenia developed after five cycles of GCD therapy, no immune-related adverse event was observed. This treatment-related adverse event was managed by reducing the gemcitabine dose to 80% and administering granulocyte colony-stimulating factor, thus enabling treatment continuation. 18F-FDG PET–CT images in pretreatment and post eight cycles of GCD therapy showed no viable lesions except in the liver (Fig. 5).

**Fig. 6:**
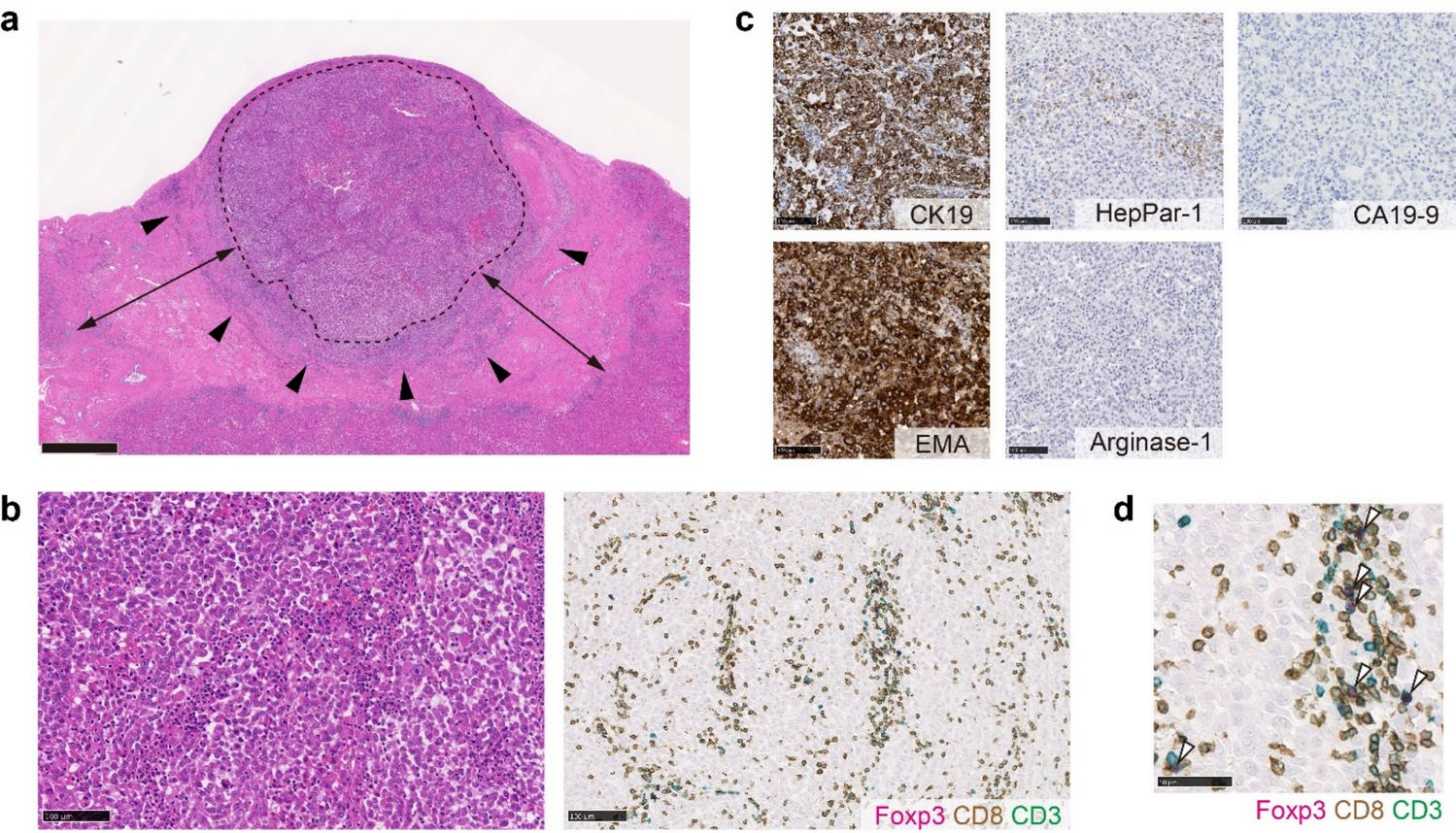
Histopathology and tumor immune microenvironment of the resected tumor after treatment. **a** Loupe image of the resected tumor. The regression bed (double arrow), tertiary lymphoid structure (arrowhead), and band-like lymphocytic infiltration are shown. Scale bar: 2 cm. **b** Tumor is composed of poorly differentiated carcinoma with increased T-cell infiltration (total T cell: 1426 cells/mm^2^, CD8 T cell: 1393 cells/mm^2^, Treg/CD8 ratio: 0.016). Scale bar: 100 μm. **c** Immunohistochemistry results of CK19, EMA, HepPar-1, arginase-1, and CA19-9. Scale bar: 100 μm. **d** Inset of (**b**) is shown. Infiltrating T cells are mainly composed of CD8 T cells with a low ratio of regulatory T cells to CD8 T cells. Scale bar: 50 μm. Arrow heads indicate Foxp3^+^CD3^+^CD8^−^ Treg cells

After two cycles of maintenance durvalumab therapy, the patient underwent partial hepatectomy of liver segment 6. A postoperative pathological examination (Fig. 6a–d) confirmed poorly differentiated carcinoma with negative surgical margins (R0 resection). The resected tumor was composed of tumor cells with increased cellular atypia compared to those observed in the pretreatment tumor biopsy, and differentiated component characterized by tubule formation was not observed. Immunohistochemistry revealed that the tumor cells were diffusely positive for CK19 and EMA, focally and weakly positive for HepPar-1, and negative for arginase-1, thus supporting its cholangiocytic phenotype.

*TIME status of resected tumor*: We further analyzed the TIME of post-treatment tumor. A regression bed characterized by dense fibrosis was observed around the residual viable tumors and associated with the formation of tertiary lymphoid structure (TLS). Increased infiltration of T cells was observed within the viable tumor tissue (total T cell: 1426 cells/mm^2^, CD8 T cell: 1393 cells/mm^2^). These infiltrating T cells were mainly CD8 T cells, and the Treg/CD8 ratio was quite low (Treg/CD8 ratio: 0.016) (Fig. 6d) when compared to the pre-treatment biopsy.

The patient was discharged 7 days postoperatively. No additional adjuvant therapy was administered, and follow-up evaluations at 3-month intervals were scheduled. Since that time, the patient has remained free of tumor recurrence.

## Discussion

We encountered a case of advanced ICC treated with GCD therapy that resulted in significant tumor shrinkage. Ultimately, conversion surgery was successfully performed, and tumor-free status was achieved. Pathological examination of pre-treatment tumor biopsy using multiplex immunohistochemistry revealed a high frequency of tumor-infiltrating T cells in this patient, which is recognized as a positive prognostic factor for the combination immunotherapy against advanced HCC. The Treg/CD8 ratio was near the median of those observed in advanced HCC [4]. These findings suggest that the assessment of TIME in pretreatment tumor biopsy could help to predict the clinical effects of combined immunotherapy for advanced ICC and may need more research.

Histopathological changes induced by chemotherapy (such as GC) and ICI therapy (such as durvalumab) both include intratumoral necrosis. In addition, the activated antitumor immune response induced by ICI eradicates tumor tissue, primarily from the tumor margins, and replaces it with dense fibrotic tissue referred to as a regression bed around viable tumor tissue [5]. This active immune response is also associated with TLS formation. After treatment of the present case, the tumor exhibited a well-defined regression bed with TLS formation and band-like lymphocytic infiltration, thus confirming that durvalumab had an additional therapeutic effect.

TIME before treatment is a crucial factor that can predict the efficacy of ICI therapy in many types of tumors. In general, increased T-cell infiltration, lower Treg frequency, and TLS formation are associated with positive prognostic outcomes after ICI treatment [4, 6, 7]. The fibrotic stroma around tumor nests contributes to the exclusion of T-cell infiltration. Studies based on whole transcriptome analysis have demonstrated that approximately 10% of resectable ICC cases comprise a TIME with increased lymphocytic infiltration and negatively correlated inflammatory and fibrotic gene signatures [8–11], and the current case may correspond to these “inflammatory” subtypes. One study has analyzed the roles of immune microenvironment in predicting the clinical effect of combined hepatocellular cholangiocarcinoma [12]. However, the characteristics of the TIME with advanced ICC cases and its relationship with the efficacy of combined immunotherapy remain unclear and require future study.

In more detail, Job et al. classified surgically resected ICCs into four immune subtypes (I1–I4) using whole transcriptome analysis [8]. The I1 “immune desert” subtype (approximately 59% of tumors) is characterized by immunologic ignorance and a lack of T cells. The I2 “immunogenic” subtype (approximately 11% of tumors) features an inflammatory phenotype with immune cells that closely interact with tumor cells within a stimulatory and suppressive TIME. The present case may be consistent with this subtype. The I3 “myeloid” subtype (approximately 13% of tumors) is dominated by M2 macrophages and CD4 + T cells, whereas the I4 “mesenchymal” subtype (approximately 17% of tumors) is associated with high levels of vascular factors and activated fibroblast-derived chemokines. From a therapeutic perspective, the I2 immunogenic subtype responds well to ICI therapies and is associated with improved survival.

Martin-Serrano et al. classified ICI-treated tumors into five immune subtypes also using whole transcriptome analysis [9]. The immune classical (approximately 10% of tumors) with which the present case was consistent and inflammatory stroma (approximately 25% of tumors) classes, which are both inflamed classes, differ in their oncogenic pathways and extent of desmoplasia; the inflammatory stroma class exhibits T-cell exhaustion, abundant stroma, and KRAS mutations. Among the noninflamed classes, the desert-like (approximately 20% of tumors) class has the lowest immune infiltration and abundant Treg cells, whereas the hepatic stem-like class (approximately 35% of tumors) is enriched with M2-like macrophages, mutations in *IDH1/2*, and *BAP1* and *FGFR2* fusions. The remaining tumor class is the classical (approximately 10% of tumors) class, which is defined by cell-cycle pathways and a poor prognosis.

In the current case, we have focused on the infiltration of total T cell, CD8 T cell, and Treg, based on the previous researches showing that these T-cell-related parameters are predictive for the clinical effect of combined immunotherapy in some types of tumors [4, 6, 7]. Since the whole landscape of TIME of advanced ICC, composed of various T and B cell subtypes, innate lymphoid cells, myeloid cells including macrophages, tumor vessels, and fibroblasts is still largely unclear, we have provisionally compared with our previous data set in the TIME of advanced HCC [4]. Therefore, it requires further research using pretreatment tumor biopsies to elucidate what type of TIME is associated with the clinical effect or resistance after combined immunotherapy against advanced ICC. Based on this future research, appropriate cutoff values for the TIME-related parameters should be determined.

Finally, in this case, we discontinued ICI therapy with the full consensus of the patient. However, there remains a possibility of tumor recurrence, since we did not resect the lung and lymph node metastases. In addition, there is no established consensus on the optimal duration for maintaining ICI therapy. Jansen et al. reported that in patients with advanced melanoma who were treated with an anti-PD-1 antibody, achieved complete remission (CR), and received treatment for more than 6 months, the risk of relapse after discontinuation was low [13]. Our patient achieved complete remission, excluding the liver lesion, after three cycles of GCD therapy, and ICI-based therapy was continued for more than 6 months.

Now, approximately 6 months have passed since the liver resection, and fortunately, no tumor recurrence has been observed. However, strict follow-up remains essential.
